# Cerebrovascular gene expression in spontaneously hypertensive rats

**DOI:** 10.1371/journal.pone.0184233

**Published:** 2017-09-07

**Authors:** Anne-Sofie Grell, Simona Denise Frederiksen, Lars Edvinsson, Saema Ansar

**Affiliations:** 1 Department of Clinical Experimental Research, Glostrup Research Institute, Rigshospitalet Glostrup, Glostrup, Denmark; 2 Division of Experimental Vascular Research, Department of Clinical Sciences, Lund University, Lund, Sweden; Universidade Federal do Rio de Janeiro, BRAZIL

## Abstract

Hypertension is a hemodynamic disorder and one of the most important and well-established risk factors for vascular diseases such as stroke. Blood vessels exposed to chronic shear stress develop structural changes and remodeling of the vascular wall through many complex mechanisms. However, the molecular mechanisms involved are not fully understood. Hypertension-susceptible genes may provide a novel insight into potential molecular mechanisms of hypertension and secondary complications associated with hypertension. The aim of this exploratory study was to identify gene expression differences in the middle cerebral arteries between 12-week-old male spontaneously hypertensive rats and their normotensive Wistar-Kyoto rats using an Affymetrix whole-transcriptome expression profiling. Quantitative PCR and western blotting were used to verify genes of interest. 169 genes were differentially expressed in the middle cerebral arteries from hypertensive compared to normotensive rats. The gene expression of 72 genes was decreased and the gene expression of 97 genes was increased. The following genes with a fold difference ≥1.40 were verified by quantitative PCR; *Postn*, *Olr1*, *Fas*, *Vldlr*, *Mmp2*, *Timp1*, *Serpine1*, *Mmp11*, *Cd34*, *Ptgs1* and *Ptgs2*. The gene expression of *Postn*, *Olr1*, *Fas*, *Vldlr*, *Mmp2*, *Timp1* and *Serpine1* and the protein expression of LOX1 (also known as OLR1) were significantly increased in the middle cerebral arteries from spontaneously hypertensive rats compared to Wistar-Kyoto rats. In conclusion, the identified genes in the middle cerebral arteries from spontaneously hypertensive rats could be possible mediators of the vascular changes and secondary complications associated with hypertension. This study supports the selection of key genes to investigate in the future research of hypertension-induced end-organ damage.

## Introduction

Hypertension is a hemodynamic disorder and one of the most important and well-established risk factors for cardiovascular diseases and stroke. Essential hypertension is defined as blood pressure of 140/90 mmHg or above of unknown cause [1]. The blood pressure is not controlled in nearly 50% of hypertensive patients due to no obvious symptoms of essential hypertension [2,3]. The consequence is a large group of patients in high risk of secondary complications. During essential hypertension, the blood vessels are exposed to chronic shear stress which leads to structural changes and remodeling of the vascular wall. Hypertension causes vascular remodeling through many different and complex mechanisms [4,5], but the underlying gene expressions are not fully understood. To gain a better understanding of these mechanisms, this exploratory study is to our knowledge the first to identify gene expression differences in the middle cerebral arteries (MCAs) between spontaneously hypertensive rats (SHRs) and their normotensive Wistar-Kyoto rats (WKY rats) using an Affymetrix whole-transcriptome expression profiling (termed whole-genome microarray gene-expression profiling). A whole-genome microarray gene-expression profiling is a useful tool to permit an unbiased selection of genetic targets of hypertension and potential secondary complications associated with hypertension. Our unique focus on the MCAs is important, since hypertension is a vascular disease and a risk factor of other cerebrovascular diseases such as stroke. It also contributes to elevated vascular resistance by changes in the vascular wall, and pial arteries such as the MCAs contribute to the cerebrovascular resistance [6]. Especially the regions of arterial branching are more susceptible to altered shear stress due to the greater effect of hemodynamic forces such as mechanical stretch [7].

Hypertension induces excessive stress on the vascular wall over time, causing alterations in the wall thickness and composition. Implicated alterations are growth and migration of smooth muscle cells (SMCs), endothelial dysfunction, inflammation, cell death and synthesis and degradation of the extracellular matrix (ECM). These alterations as well as humoral factors modify the mechanical and hemodynamic properties of the arteries [5]. It is acknowledged, that the alterations for example enhance the wall thickness, reduce the lumen of the artery or lead to arterial stiffening [8]. As a consequence of all these hypertension-induced changes in the vascular wall, the genes and their transcriptomic products are up- or downregulated.

The aim of this exploratory study was to investigate genes involved in the vascular changes associated with hypertension. This study contributes to the current knowledge of the molecular mechanisms that are involved in hypertension and in the potential secondary complications associated with hypertension.

## Materials and methods

### Ethics

All experiments were carried out in strict accordance with the guidelines from the European Community Council directive (2010/63/EU) for Protection of Vertebrate Animals Used for Experimental and other Scientific Purposes and were approved by the Danish Animal Experiments Inspectorate (Permit Number: 2014-15-0201-00042). The study complies with the ARRIVE guidelines (Animal Research: Reporting *In Vivo* Experiments). All surgeries were performed under isoflurane anesthesia, and all efforts were made to minimize suffering.

### Animals

12-week-old male SHRs (257-311g) and their normotensive control WKY male rats (299-340g) were obtained from Charles River (Charles River laboratories, Sulzfeld, Germany). The SHR is an animal model of essential hypertension [9] and develops hypertension at 4 weeks of age [10] why this animal model can provide useful information and implications of pathophysiological processes in humans. The rats were housed with a 12 hours light/dark cycle and provided with standard rat chow and tap water *ad libitum*.

### Blood pressure measurement and harvest of arteries

The rats were anaesthetized with 2.5% isoflurane in N_2_O/O_2_ (70:30) followed by cannulation of the tail artery to record the MABP for five minutes. This is a direct measurement of the blood pressure [11]. The rats were sacrificed by decapitation, and the MCAs were carefully dissected from the brain and stripped of connective tissue and blood in ice-cold sodium Krebs buffer (NaCl 119mM, NaHCO_3_ 15mM, KCl 4.6mM, MgCl_2_ 1.2mM, NaH_2_PO_4_ 1.2mM, CaCl_2_ 1.5mM and glucose 5.5mM) oxygenated with 5% CO_2_ in O_2_. The tissue was snap frozen on dry ice and kept at -80°C until the preparation of RNA extraction.

### RNA preparation

Total RNA extraction was prepared in the same way for the whole-genome microarray gene-expression profiling and quantitative PCR (qPCR). Tissue from one MCA was homogenized on dry ice 3x20sec in lysis buffer (ML buffer) from the NucleoSpin miRNA isolation kit (Macherey-Nagel, Germany) using a FastPrep-24^™^ 5G instrument (MP Biomedicals, USA). Lysing matrix D tubes containing 1.4mm ceramic spheres (MP Biomedicals, USA) were used for tissue homogenization. Total RNA extraction was carried out using the NucleoSpin miRNA isolation kit according to the manufacturers’ protocol. The RNA was eluted in 30μl RNAse free water. Total RNA concentration was measured using the NanoDrop 2000 UV-Vis spectrophotometer (ThermoFisher Scientific, MA, USA), whereupon a ratio of sample absorbance at 260nm and 280nm in the range of 1.7 to 2.1 was acceptable. The integrity of the RNA was measured using the Agilent 2100 Bioanalyzer (Agilent Technologies, CA, USA), where the acceptable RNA integrity number was ≥7.

### Whole-genome microarray gene-expression profiling

Affymetrix whole-transcriptome expression profiling was processed by Swegene centre for integrative biology (SCIBLU) genomics, Affymetrix unit at Lund University, Sweden.

A total of 100ng RNA was primed with primers containing a T7 promoter sequence to synthesize first-strand cDNA. The single-stranded cDNA was then converted to a double-stranded cDNA and used as a template for the *in vitro* transcription (IVT) to synthesize antisense RNA (also called complimentary RNA or cRNA) using a T7 RNA polymerase. This procedure is known as the Eberwine or RT-IVT method [12]. The cRNA was purified by removing enzymes, salts, inorganic phosphates and unincorporated nucleotides and quantified for the 2^nd^-cycle single-stranded cDNA synthesis, from where sense-strand cDNA (ss-cDNA) was synthesized. RNase H was used to hydrolyze the cRNA template leaving ss-cDNA. After hydrolysis, the ss-cDNA was purified to remove enzymes, salts and unincorporated dNTPs tfor fragmentation and labelling. ss-cDNA was fragmented by uracil-DNA glycosylase and apurinic/apyrimidinic endonuclease-1 at the unnatural dUTP residues that breaks the DNA strand. The fragmented ss-cDNA was labelled by terminal deoxynucleotidyl transferase using the Affymetrix proprietary DNA Labelling Reagent that is covalently linked to biotin allonamide triphosphate. The fragmented and biotin-labelled ss-cDNA was added to a hybridization cocktail onto the Affymetrix GeneChip rat gene 2.0 ST arrays followed by hybridization for 16 hours at 45°C in an Affymetrix Gene Chip Hybridization 645 oven. The array was washed and stained on the GeneChip Fluidics Station 450 using the appropriate fluidics script before being inserted onto an Affymetrix autoloader carousel and scanned using the Affymetrix GeneChip scanner 3000 7G.

### cDNA synthesis and qPCR

66ng RNA was synthesized to cDNA using the RT^2^ First Strand Kit (Qiagen, USA) according to the manufacturer’s protocol. qPCR was performed in a 10μl reaction volume containing TaqMan 2x universal PCR master mix (ThermoFisher Scientific, MA, USA), 20x TaqMan gene expression assay, RNAse free water and 2μl cDNA using the QuantStudio 12K Flex real-time PCR system (ThermoFisher Scientific, MA, USA) with ROX as a passive reference. A no-template control with RNAse free water instead of cDNA was used as negative control for all TaqMan gene expression assays. An inter-plate control for all TaqMan gene expression assays was used to control the thermal cycling between plates. All TaqMan gene expression assays were pipetted in triplicates for each sample. Taqman gene expression assays, for the following genes, were purchased from ThermoFisher Scientific, MA, USA: periostin, osteoblast specific factor (*Postn*) (Rn01494627_m1), oxidized low density lipoprotein (lectin-like) receptor 1 (*Olr1*) (Rn00591116_m1), fas, TNF receptor superfamily member 6 (*Fas*) (Rn00685720_m1), very low density lipoprotein receptor (*Vldlr*) (Rn01498167_m1), matrix metallopeptidase 2 (*Mmp2*) (Rn01538170_m1), tissue inhibitor of metallopeptidase 1 (*Timp1*) (Rn00587558_m1), serpin peptidase inhibitor, clade E (nexin, plasminogen activator inhibitor type 1, member 1 (*Serpine1)* (Rn01481341_m1), cd34 molecule (*Cd34*) (Rn_03416140_m1), prostaglandin-endoperoxide synthase 1 (*Ptgs1*) (Rn00566881_m1), *Ptgs2* (Rn01483828_m1), *Mmp11* (LOC103694874), glyceraldehyde-3-phosphate dehydrogenase (*Gapdh*) (Rn01749022_g1), actin beta (*Actb*) (Rn00667869_m1). *Gapdh* and *Actb* were used as reference genes to normalize the mRNA levels. The thermal cycling condition included an initial denaturation step at 50°C for 2min and 95°C for 10min followed by 45 PCR cycles at 95°C for 15sec and 60°C for 1min.

### Western blotting

Cerebral arteries from two rats were pooled and sonicated 3x20 pulses (output 30) on ice in RIPA buffer containing phosphatase inhibitor (Sigma-Aldrich) and protease inhibitor (Sigma-Aldrich). For further denaturation the samples were kept at -80°C for 30 min and then centrifuged at 14.000rpm for 10min at 4°C. The protein concentration in the supernatant was determined using the DC protein assay kit II (Bio-Rad) with a protein standard II (Bio-Rad) of bovine serum albumin as a standard curve. The samples were measured on the Infinite M200 (Tecan) with a wavelength of 750nm. 14μg protein was calculated according to the protein concentration, and LDS buffer (Expedeon), DTT (Expedeon) and milliQ water were added before the samples were boiled at 95°C for 5 min. The samples were loaded on a 4–20% SDS precast gel (Expedeon) and run at 180V for 70 min. The proteins were transferred to a nitrocellulose membrane (GE Healthcare, Amersham 0.2 NC) at 150V and 350mAmp for 70 min, whereupon the membrane was blocked for unspecific binding for one hour at room temperature in TBS-T buffer (TBS + 1% Tween 20) containing 2% ECL prime blocking agent (GE Healthcare, Amersham). The membrane was incubated over night at 4°C with rabbit polyclonal anti-lectin-like oxidized low density lipoprotein receptor 1 (LOX1) antibody (Abcam, ab60178, Lot number GR159762-5, synthetic peptide near the N-terminus of human Lox-1, AB_943982) diluted 4:1000 (LOX1 is also known as OLR1). Jurkat cells (gift from senior researcher Birgitte Rahbek Kornum, Department of Clinical Biochemical, Glostrup Research Institute, Rigshospitalet Glostrup) were used as a running control, and monoclonal anti-β-Actin-Peroxidase produced in mouse (Sigma A3854, batch number 026M4820V, N-terminal sequence: Ac-Asp-Asp-Asp-Ile-Ala-Ala-Leu-Val-Ile-Asp-Asn-Gly-Ser-Gly-Lys conjugated to KLH, AB_262011) diluted 1:50.000 was used as a reference antibody.

The following day the membrane was incubated with a horseradish peroxidase (HRP) conjugated secondary antibody for one hour at room temperature; anti-rabbit IgG HRP (Cell Signaling #7074) diluted 1:2000. The membrane was stripped (ReBlot Plus Strong from Millipore) for 8min at room temperature, blocked and incubated one hour at 4°C with the β-ACTIN antibody. This antibody was developed with no secondary antibody, since it already contains HRP.

All antibodies were diluted in TBS-T buffer containing 2% ECL prime blocking agent.

Proteins were detected using the ECL select western blotting detection reagent (GE Healthcare, Amersham) and visualized in a Fujifilm LAS-4000 Luminiscent Image Analyser.

### Statistical methods

#### Blood pressure and body weight

In total 35 rats (WKY, n = 17 and SHR, n = 18) were used for the whole-genome microarray gene-expression profiling, qPCR and western blotting. No power calculation was used to determine sample size. A two-tailed Mann-Whitney test was used for the statistical analysis of MABP and body weight using GraphPad Prism 5. Data is expressed as median ± interquartile range (IQR). P-value <0.05 is considered statistical significant.

#### Whole-genome microarray gene-expression profiling

In total 10 rats (WKY rat, n = 5 and SHR, n = 5) were used for the whole-genome microarray gene-expression profiling. Basic Affymetrix GeneChip and experimental quality analyses were performed using the Expression Console Software v1.1.2, and the Robust Multi-array analysis method was used for probe summarization and data normalization (quantile normalization and log transformation) using v1.4.1.46. Data filtration was done for probe sets having a value less than the median values of the negative control in 80% of total samples. Significance analysis of microarrays (SAM) was performed using the TMEV v4.0 software to identify significantly differentially expressed genes between groups (q = 0, termed differentially expressed genes) [13]. The q-values represent False Discovery Rate (FDR) adjusted p-values, for which q = 0 denotes the statistical significant level. The fold difference (FD) is a measure describing how much a quantity changes going from an initial to a final value. If the FC is positive, the gene expression is increased in the MCAs from SHRs compared to WKY rats, and if it is negative, the gene expression is decreased in the MCAs from SHRs compared to WKY rats.

#### Gene ontology (GO) overrepresentation analysis

All differentially expressed genes (q = 0) in the MCAs from SHRs compared to WKY rats were included in the GO overrepresentation analysis. Information of genes annotated to the ontologies was extracted from the GO database using BiomaRt v2.26.1 [14,15]. GO terms with ≤5 annotated genes were excluded from the analysis. P-values for each GO term were calculated by a right-tailed Fisher’s exact tests for count data and adjusted for multiple testing. GO terms with FDR-adjusted p-values <0.05 are considered statistical significant.

#### qPCR

In total 13 rats (WKY rat, n = 6 and SHR, n = 7) were used for the qPCR experiments. These rats were not the same rats used in the whole-genome microarray gene-expression profiling. The threshold cycle (C_t_) values refer to the number of PCR cycles, where the hydrolysis probe begins to fluoresce. C_t_ values were determined using the QuantStudio 12K Flex software (ThermoFischer Scientific, MCA, USA), and the technical triplicates were averaged. Automatic background fluorescence, thresholds of the samples, no-template control and inter-plate control were checked. Adjustment of the averaged C_t_ values of each TaqMan gene expression assays were adjusted relative to its inter-plate control. ΔC_t_ (*C*_*t*, *sample*_ − *C*_*t*, *average of references*_) values are plotted on the y-axe in the graphs by a logarithmic scale. The figures are presented as a scatter plot expressed as median ± IQR, and *n* represents to the number of rats. A two-tailed Mann-Whitney test was used for the statistical analysis using GrapPad Prism 5. P-value <0.05 is considered statistical significant.

#### Western blotting

In total 12 rats were used for the western blotting experiments. The cerebral arteries from two rats were pooled into one sample (WKY rat, n = 3 and SHR, n = 3). The samples were run twice at different days and normalized to Jurkat cells and β-ACTIN. A two-tailed Mann-Whitney test was used for the statistical analysis using GraphPad Prism 5. Data is expressed as median ± IQR, and *n* represents the number of rats. P-value <0.05 is considered statistical significant.

## Results

### Blood pressure and body weight

MABP was significantly higher in 12-week-old SHRs (155 (147–164) mmHg) compared to the age-matched WKY rats (108 (103–114) mmHg) (P<0.0001), and the body weight was significantly lower in SHRs (279 (263–292) g) compared to WKY rats (312 (309–332) g) (P<0.0001).

### Whole-genome microarray gene-expression profiling

**Fig 1:**
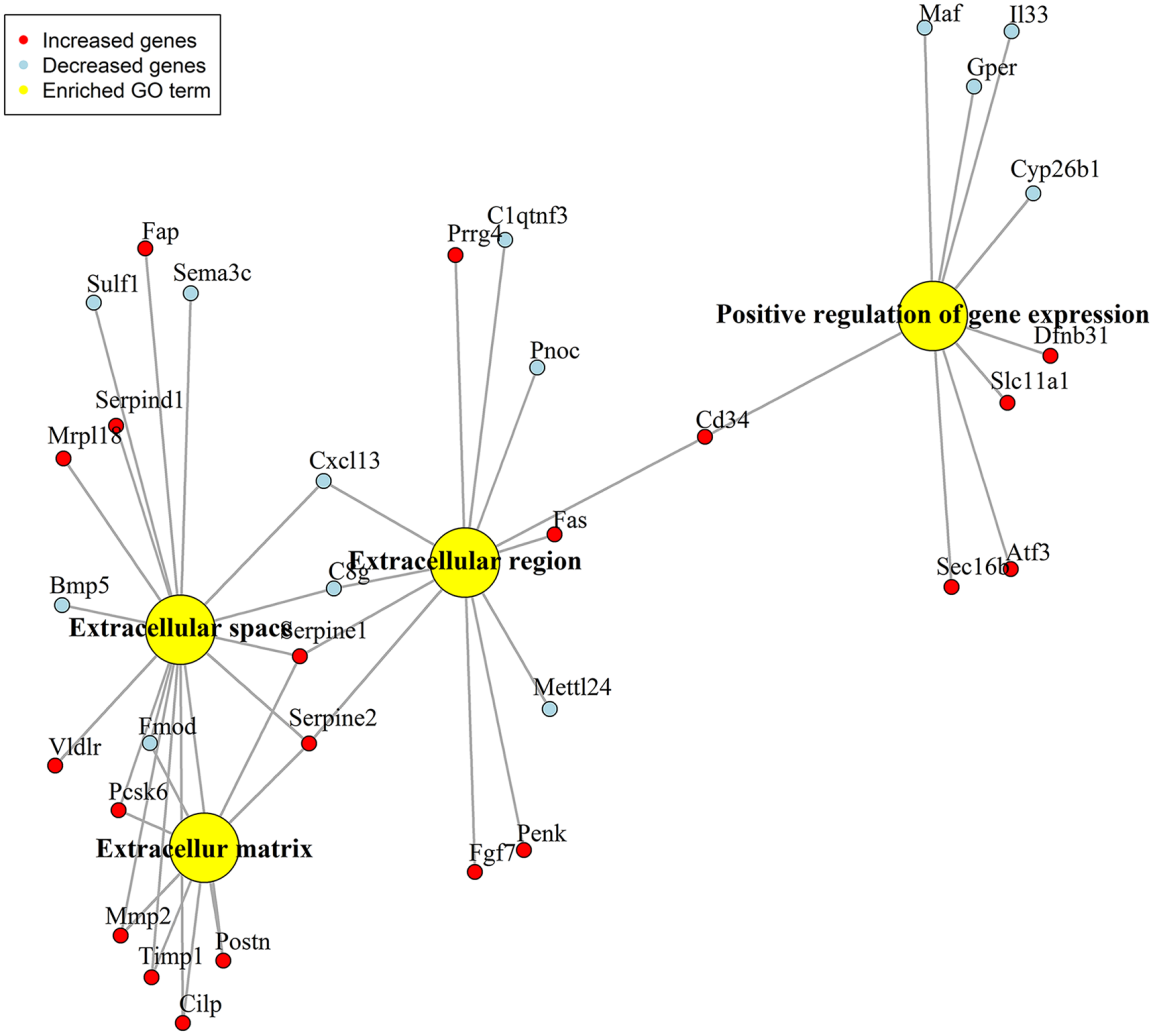
GO overrepresentation analysis network. Network of the differentially expressed genes (q = 0) in the MCAs from SHRs (n = 5) compared to WKY rats (n = 5).

In total 14,150 probe-set IDs with a gene accession number were detected, and 169 genes were differentially expressed (q = 0) in MCAs from SHRs compared to WKY rats (S1 Table). Out of 169 genes, the expression of 72 genes was decreased and the expression of 97 genes was increased in MCAs from SHRs compared to WKY rats. Using the GO overrepresentation analysis, all the differentially expressed genes (169 genes) were classified into two GO ontologies named biological process and cellular component. Biological process was further subdivided into one GO term named positive regulation of gene expression, and cellular component was further subdivided into three GO terms named extracellular region, extracellular matrix and extracellular space (Fig 1).

We decided to proceed with the genes from the GO ontology named cellular component with a FC ≥1.40 (16 individual genes) (Table 1), since they are related to the extracellular part of the MCAs and might be implicated in the vascular changes associated with hypertension.

**Table 1:** Differentially expressed genes from the GO ontology named cellular component with a fold difference ≥1.40.

GO name (GO id)	Adjusted P-value (FDR)	Gene	Description	FD
Extracellular matrix (GO:0031012)		*Postn*	Periostin, osteoblast specific factor	2.634
		*Cilp*	Cartilage intermediate layer protein, nucleotide pyrophosphohydrolase	2.215
	0.0017	*Serpine1*	Serpin peptidase inhibitor, clade E (nexin, plasminogen activator inhibitor type 1), member 1	1.835
		*Timp1*	TIMP metallopeptidase inhibitor 1	1.588
		*Pcsk6*	Proprotein convertase subtilisin/kexin type 6	1.533
		*Serpine2*	Serpin peptidase inhibitor, clade E, member 2	1.482
		*Mmp2*	Matrix metallopeptidase 2	1.465
Extracellular region (GO:0005576)		*Penk*	Proenkephalin	2.155
		*Serpine1*	Serpin peptidase inhibitor, clade E (nexin, plasminogen activator inhibitor type 1), member 1	1.835
	0.0244	*Fgf7*	Fibroblast growth factor 7	1.780
		*Cd34*	Cd34 molecule	1.658
		*Fas*	Fas (TNF receptor superfamily, member 6)	1.550
		*Serpine2*	Serpin peptidase inhibitor, clade E, member 2	1.482
		*Prrg4*	Proline rich Gla (G-carboxyglutamic acid) 4 (transmembrane)	1.430
Extracellular space (GO:0005615)		*Postn*	Periostin, osteoblast specific factor	2.634
		*Cilp*	Cartilage intermediate layer protein, nucleotide pyrophosphohydrolase	2.215
		*Fap*	Fibroblast activation protein, alpha	1.956
		*Serpine1*	Serpin peptidase inhibitor, clade E (nexin, plasminogen activator inhibitor type 1), member 1	1.835
	0.0488	*Serpind1*	Serpin peptidase inhibitor, clade D (heparin cofactor), member 1	1.772
		*Timp1*	TIMP metallopeptidase inhibitor 1	1.588
		*Mrpl18*	Mitochondrial ribosomal protein L18	1.536
		*Pcsk6*	Proprotein convertase subtilisin/kexin type 6	1.533
		*Serpine2*	Serpin peptidase inhibitor, clade E, member 2	1.482
		*Mmp2*	Matrix metallopeptidase 2	1.465
		*Vldlr*	Very low density lipoprotein receptor	1.440

GO, gene ontology; FDR, false discovery rate; FD, fold difference.

More specific, we selected *Fas* and *Cd34* from the GO term named extracellular region and *Mmp2*, *Serpine1*, *Timp1* and *Postn* from the GO term named extracellular matrix and *Vldlr* from the GO term named extracellular space. *Mmp2*, *Timp1* and *Postn* were also expressed in the GO term named extracellular space and *Serpine1* in the GO term named extracellular region. This is another reason, why we proceeded with the GO ontology named cellular component.

Additional to the GO overrepresentation analysis, the putative function of the genes with a FC ≥1.40 (90 genes) from the whole-genome microarray gene-expression profiling was investigated by a literature search using PubMed. Apart from *Mmp11* which is a matrix metalloproteinases as *Mmp2* we selected *Ptgs1*, *Ptgs2* and *Olr1* due to their possible relation to the vascular changes associated with hypertension.

**Fig 2:**
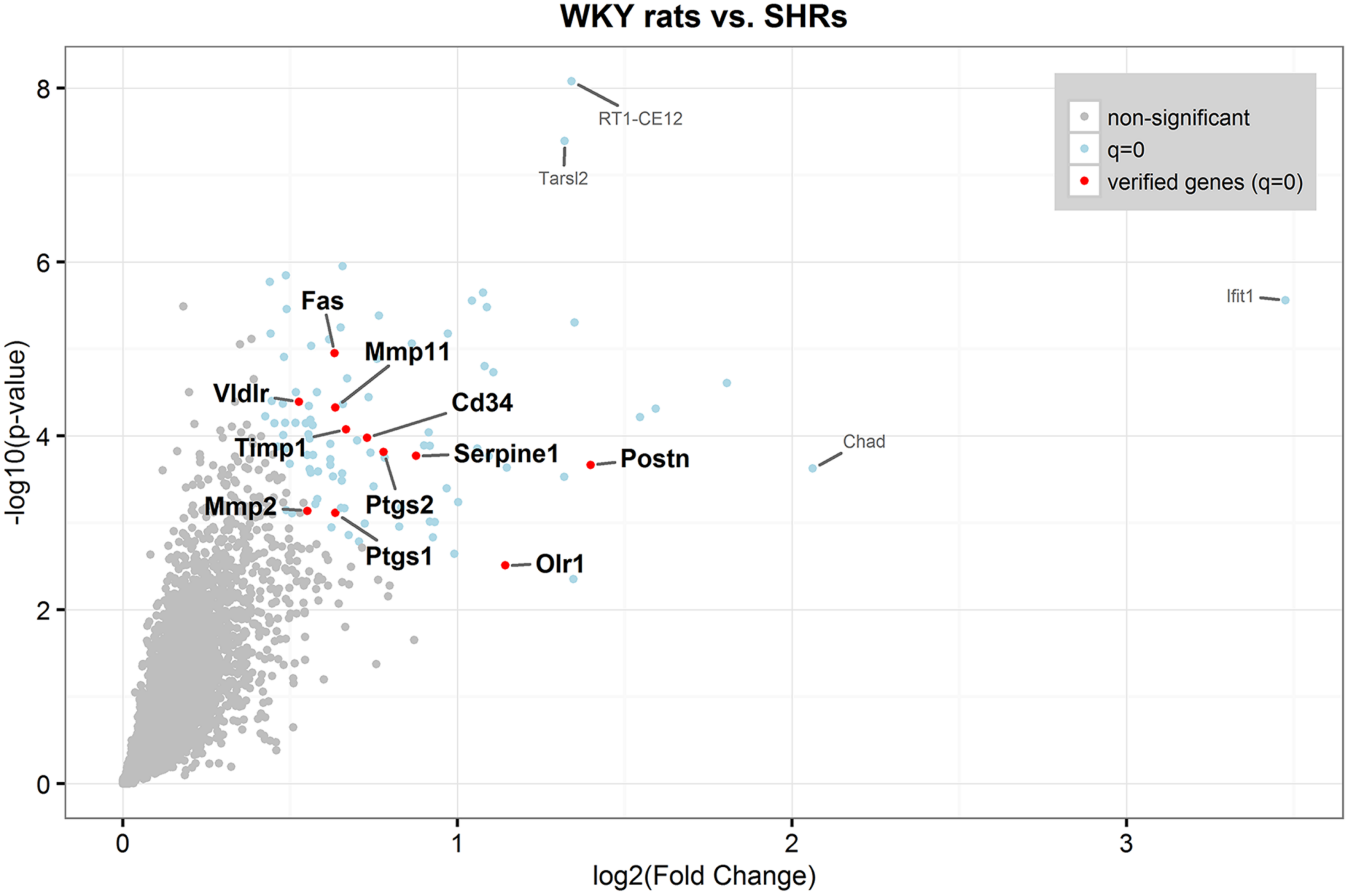
Volcano plot. Red and blue dots are differentially expressed genes (q = 0) in the MCAs from SHRs (n = 5) compared to WKY rats (n = 5). Red dots are genes verified by qPCR.

**Fig 3:**
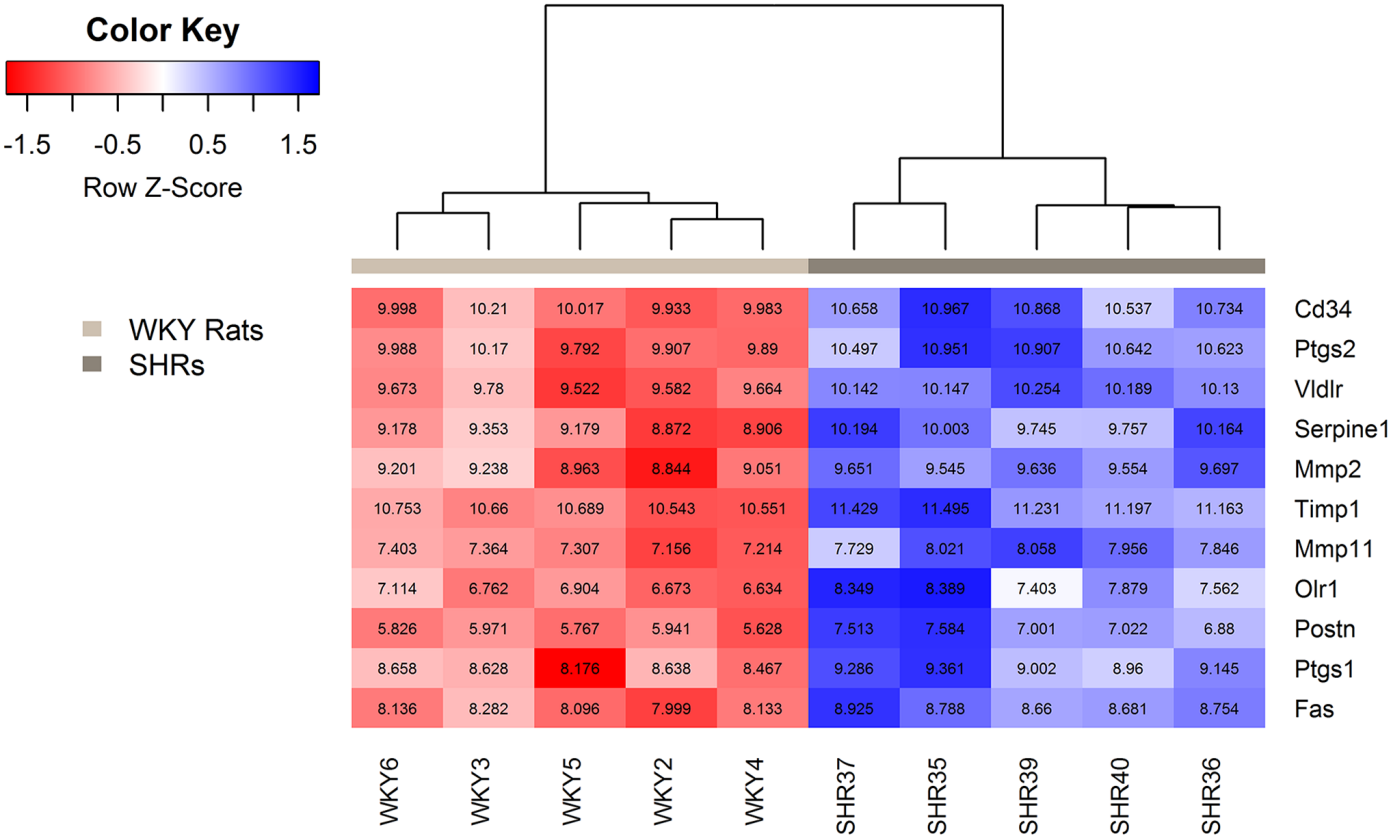
Heat map of genes verified by qPCR. Box color and color key show the expression level differences between WKY rats (n = 5) and SHRs (n = 5), reported as the Z score, which is the scaled gene expression measurement (scaled row-wise, mean = 0, SD = 1). As expected, WKY rats and SHRs are grouped separately due to distinct expression patterns between groups. The numbers in the boxes represent normalized expression values of each gene for each rat.

The eleven genes verified by qPCR are illustrated in a volcano plot in Fig 2 and in a heat map in Fig 3.

#### qPCR

We examined the gene expression of *Gapdh* and *Actb* to verify, that the reference genes were stable between groups. The C_t_ values between SHRs and WKY rats for both genes were not statistical significant (*Gapdh*: SHRs, 25.8 (25.7–26.0); WKY rats 25.9 (25.7–26.0), P = 0.2662. *Actb*: SHRs, 23.6 (23.1–23.8); WKY rats 23.5 (23.1–23.8), P = 0.6109).

We decided to verify differentially expressed genes with a FC ≥1.40 in MCAs from SHRs compared to WKY rats due to a low template concentration, and to be sure to detect the gene expression above the detection limit (C_t_ value >35). The following eleven genes were chosen for verification with qPCR: *Postn*, *Olr1*, *Fas*, *Vldlr*, *Mmp2*, *Timp1*, *Serpine1*, *Mmp11*, *Cd34*, *Ptgs1* and *Ptgs2* (S2 Table). The verification was done on different RNA preparations than used in the whole-genome microarray gene-expression profiling to verify the technical process and the biological variation. The ΔC_t_ values of *Postn*, *Olr1*, *Fas*, *Vldlr*, *Mmp2*, *Timp1* and *Serpine1* were significantly increased in MCAs from SHRs compared to WKY rats (S1 Fig, Table 2). There was no change in the ΔC_t_ values of *Cd34*, *Ptgs2* and *Mmp11* between the MCAs from SHRs and WKY rats (S2 Fig, Table 2). A reason for this could be the big variation between the samples in both the WKY and SHR group.

**Table 2:** Expression levels of genes verified by qPCR.

Gene	WKY rats	SHRs	P-value
	Median (IQR)	Median (IQR)	
*Postn*	7.5 (7.3–8.2)	6.1 (5.8–6.8)	0.0159
*Olr1*	10 (9.8–10.3)	7.5 (6.4–7.7)	0.0079
*Fas*	5.7 (5.6–6.0)	5.2 (5.1–5.4)	0.0079
*Vldlr*	6.0 (5.7–6.4)	4.9 (4.6–5.3)	0.0079
*Mmp2*	6.4 (6.3–6.6)	5.8 (5.7–6.3)	0.0247
*Timp1*	5.7 (5.6–6.0)	5.1 (5.0–5.2)	0.0022
*Serpine1*	5.2 (5.1–5.4)	3.6 (3.5–3.8)	0.0079
*Cd34*	4.9 (4.4–5.2)	4.9 (4.5–5.3)	1.0000
*Ptgs2*	6.2 (5.9–6.7)	5.6 (5.5–6.2)	0.3095
*Mmp11*	8.2 (8.0–8.3)	8.1 (7.8–8.7)	0.8726

IQR, interquartile range.

The *Ptgs1* gene was not expressed above the detection limit, and it was not possible to increase the template concentration (data not shown). Of the eleven genes verified by qPCR, seven genes (*Postn*, *Olr1*, *Fas*, *Vldlr*, *Mmp2*, *Timp1* and *Serpine 1*) had similar gene expression pattern as in the whole-genome microarray gene-expression profiling. These genes will be discussed according to their relation to the vascular changes associated with hypertension.

### Western blotting

**Fig 4:**
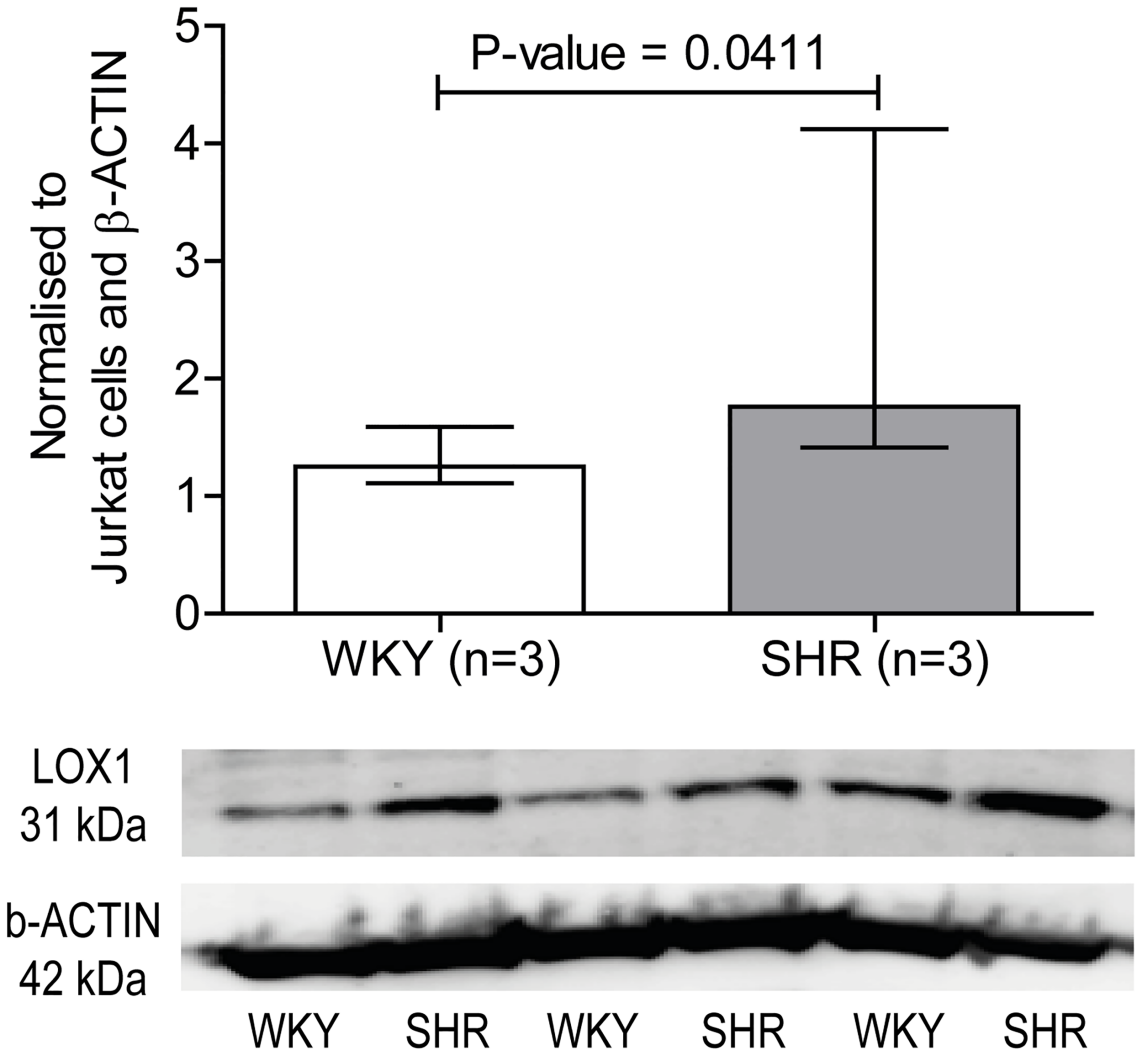
LOX1 protein level. LOX1 (also known as OLR1) expression in the cerebral arteries from SHRs compared to WKY rats and representative blot of LOX1 and β-ACTIN. Data is normalized to Jurkat cells and β-ACTIN. Data is expressed as median ± interquartile range, and *n* represents the number of rats. P-value <0.05 is considered statistical significant.

Since *Olr1* is an important scavenger receptor with a functional role in hypertension and stroke, the protein level was examined. In addition, it was one of the genes verified by qPCR with the lowest p-value (P-value = 0.0079) between the MCAs from SHRs and WKY rats. The result revealed, that LOX1 protein level was significantly increased in cerebral arteries from SHRs (1.8 (1.4–4.1)) compared to WKY rats (1.2 (1.1–1.6)) (Fig 4, S3 Fig).

## Discussion

In this study, we used a broader approach to understand the molecular mechanisms of the vascular changes in the MCAs due to hypertension by the whole-genome microarray gene-expression profiling. We demonstrated an increased gene expression of *Postn*, *Fas*, *Vldlr*, *Mmp2*, *Timp1*, *Serpine1* and *Olr1* as well as an increased LOX1 protein expression in the MCAs from hypertensive rats (hypertensive cerebral arteries) compared to normotensive rats (normotensive cerebral arteries). Vascular morphology of the cerebral arteries is already altered in the early stage of hypertension [16]. According to the GO overrepresentation analysis *Postn*, *Fas*, *Vldlr*, *Mmp2*, *Timp1* and *Serpine1* are related to the extracellular part of the hypertensive cerebral arteries. Changes in the ECM components of the vascular wall have previously been demonstrated in hypertension, since the cells synthesize the ECM to provide structural support to the artery [17]. An excess of ECM products have also been shown in other vascular diseases such as stroke [18]. *Olr1* was also found to be related to the vascular changes associated with hypertension. In the cerebral arteries, a correlation between the following genes and the vascular changes besides the secondary complications associated with hypertension is therefore suggested.

We demonstrated an increased gene and protein expression of the scavenger receptor Olr1 (also known as the lectin-like oxidized low density lipoprotein receptor 1 (Lox1)) in the hypertensive cerebral arteries which correlates with previous findings [19,20]. The basal expression of Olr1 in the vascular wall is usually very low, but it can be induced by pro-inflammatory and mechanical stimuli such as hypertension [21]. The same is suggested by our results due to the basal *Olr1* expression in normotensive cerebral arteries and the increased expression in hypertensive cerebral arteries. The receptor is primarily found to bind, internalize and degrade oxidized LDL (oxLDL) which is a mechanism linked to an inflammatory response [21]. Hypertension and stroke are both associated with increased levels of inflammatory markers reflecting the inflammatory process in both pathologies [22,23]. Polymorphism of the Olr1 gene has also been associated with the risk of developing left ventricular hypertrophy in patients with essential hypertension, and hypertrophic remodeling of the vascular wall is a characteristic for hypertension [24]. The increased *Olr1* expression in hypertensive cerebral arteries might indicate vascular remodeling and inflammation which also suggest a link between hypertension and the risk of developing secondary complications such as stroke. This is supported by clinical data, since Olr1 was found in early atherosclerotic plaque from ischemic patients [25] and correlated to the risk of having a stroke using a meta-analysis [26].

Interestingly, Olr1 has been suggested to increase Mmp2 expression in human umbilical vein endothelial cells [27] indicating a connection between their increased gene expression in hypertensive cerebral arteries.

The occurrence of *Postn* in the ECM, by the GO overrepresentation analysis, can be explained by its role as a soluble ECM protein. It mediates proliferation and migration of SMCs, and Postn expression has previously been correlated with the proliferative state of neointimal SMCs in the balloon-injured vascular wall [28]. The gene is also interesting in a clinically perspective, since Postn expression was increased in lung tissue from patients with pulmonary arterial hypertension [29]. These data combined suggests that the increased *Postn* expression in hypertensive cerebral arteries indicates SMC proliferation and thereby vascular changes in response to hypertension.

Increased *Mmp2* expression in hypertensive cerebral arteries may induce vascular changes by degrading proteins of the ECM [4]. A connection between increased Mmp2 expression and vascular remodeling of the aorta has previously been suggested in hypertensive rats [30]. Mmp2 is secreted constitutively from vascular SMCs [31] which explains the basal gene expression in normotensive cerebral arteries. The increased gene expression in hypertensive cerebral arteries is supported by an increased expression and activation of Mmp2 by mechanical stretch such as hypertension in human aortic SMCs [32]. However, the plasma level of Mmp2 from hypertensive patients has been demonstrated to be both increased and decreased [33,34], why its specific role in hypertension is yet to be elucidated.

On the other hand, remodeling of ECM by Mmps affects the migration and proliferation of SMCs and thereby the formation of a neointima [31,35] that ultimately occlude the affected arteries. Mmp2 expression has also been confirmed in ischemic brain tissue [36]. These data suggests a link between changes in the ECM and SMCs in response to hypertension and the susceptibility to secondary complications.

Mmp2 secretion was reported to be induced by increased Postn expression [37] which could be a connection between the increased expression of both genes in hypertensive cerebral arteries. Their role in the ECM modulation and SMC migration and proliferation may indicate vascular remodeling in response to hypertension that entails end-organ damage.

The increased *Timp1* expression in hypertensive cerebral arteries could be a mechanism to counterbalance the increased *Mmp2* expression, due to its role as Mmp inhibitor [38]. Timps do not have a high specificity for any particular Mmps, but it is suggested that Timp2 preferential binds Mmp2, and Timp1 binds Mmp9 [39]. This might explain why the gene expression of Mmp9 is not increased in hypertensive cerebral arteries. However, in both hypertensive and normotensive cerebral arteries the *Timp1* expression (SHRs, 5.1 (5.0–5.2); WKY rats 5.7 (5.6–6.0)) was significantly increased compared to the *Mmp2* expression (SHRs, 5.8 (5.7–6.3), P = 0.0022; WKY rats 6.4 (6.3–6.6), P = 0.0087) suggesting that Timp1 might suppress the Mmp2 activity. The correlation between Timp1 and Mmp2 expression could also be a reason for the discrepancy between the measured plasma levels of Mmp2 from hypertensive patients.

On the other hand, Mmp2 is a member of the gelatinases which digest collagen in the subendothelial basement membrane [4]. Suppressed Mmp2 expression by Timp1 could lead to increased collagen accumulation and thereby vascular remodeling of the hypertensive cerebral arteries. In a systematic review and meta-analysis, only Timp1 levels were greater in hypertensive than normotensive patients, whereas both Timp1 and Mmp2 levels were greater in hypertensive patients with heart failure than hypertensive patients without heart failure. It was therefore suggested, that both Timp1 and Mmp2 were potential plasma biomarkers of cardiovascular remodeling in response to hypertension [38].

The increased *Serpine1* expression in hypertensive cerebral arteries might be a protecting mechanism against ECM proteolysis, since Serpine1 (also known as plasminogen activating inhibitor type 1) inhibits plasminogen activators such as urokinase-type plasminogen activator (uPA). The plasminogen activators converts plasminogen to plasmin which is essential for ECM degradation and activation of the fibrinolysis [40]. Consequently, Serpine1 plays a role in maintaining the fibrin blood clot and the risk of having a stroke [41,42]. In relation to its role in hypertension and ECM degradation, Serpine1 augmented intima-media thickness contributing to endothelial dysfunction in carotid arteries from hypertensive patients [43]. This could also be a reason for the increased *Serpine1* expression in hypertensive cerebral arteries.

Vldlr was previously found to play a role in intimal thickening [44] and to regulate uPA-Serpine1 complexes [45], which could be a connection between the increased expression of both genes in the hypertensive cerebral arteries.

Fas is a cell surface death receptor, that induces SMCs apoptosis [46]. *Fas* is therefore suggested to be an apoptotic marker in the hypertensive cerebral arteries. Apoptosis is implicated in different vascular pathologies such as hypertension [47,48], and has been associated with vascular remodeling of mesenteric arteries from hypertensive rats with the same age as the rats in our study [49]. This study is to our knowledge the first to demonstrate increased *Fas* expression in hypertensive cerebral arteries, but it has also been demonstrated in brain tissue after ischemic stroke [50,51]. Altogether, *Fas* might be an indicator of hypertension-induced end-organ damage.

Interestingly, a connection between Fas and Olr1 has been suggested, since Olr1 was shown to facilitate an oxLDL-induced augmentation of Fas-mediated apoptosis [52]. This correlates with the increased expression of the genes in the hypertensive cerebral arteries.

## Conclusions

This exploratory study is the first to reveal that *Postn*, *Fas*, *Vldlr*, *Mmp2*, *Timp1*, *Serpine1* and *Olr1* could be possible genetic mediators of the vascular changes in hypertensive cerebral arteries. Interestingly, previous research supports a connection between several of the verified genes and the vascular changes associated with hypertension which highlights the importance of the reported hypertension-susceptible genes to the current knowledge of molecular mechanisms during hypertension. The strength of this study is that it is conducted in hypertensive cerebral arteries since hypertension contributes to vascular changes and is a risk factor of other cerebrovascular diseases such as stroke. Some of the genes have previously been implicated in secondary complications to hypertension. This study supports the selection of key genes to investigate in the future research of hypertension-induced end-organ damage.

## Supporting information

S1 Fig Genes with increased expression in MCAs from hypertensive compared to normotensive rats. Scatter plot of (A) *Postn*, (B) *Olr1*, (C) *Fas*, (D) *Vldlr*, (E) *Mmp2*, (F) *Timp1* and (G) *Serpine 1* expression from SHRs and WKY rats. ΔC_t_ values are plotted on the y-axe by a logarithmic scale. Data is expressed as median ± interquartile range, and *n* represents the number of rats. P-value <0.05 is considered statistical significant.
(TIF)

S2 Fig Genes with no expressional changes in MCAs between hypertensive and normotensive rats. Scatter plot of (A) *Cd34*, (B) *Ptgs2* and (C) *Mmp11* expression in middle cerebral arteries from SHRs and WKY rats. ΔC_t_ values are plotted on the y-axe by a logarithmic scale. Data is expressed as median ± interquartile range, and *n* represents the number of rats. P-value <0.05 is considered statistical significant.
(TIF)

S3 Fig Western blot membrane of LOX1 and β-ACTIN. Western blot membrane with (A) LOX1 and (B) β-ACTIN. The molecular marker is marked with a black square. Before incubation with primary antibodies the membrane was cut just before 71 kDa and 28 kDa. Sample in lane 2 and 3 are samples used to test different antibodies for the other membrane pieces. (A) According to Abcam, LOX1 proform was detected at 50 kDa and LOX1 mature form was detected at 31 kDa. However, the LOX1 proform in the antibody images from Abcam homepage seems to be at 45 kDa and not 50 kDa according to the molecular marker which correlate with the band around 48 kDa on our membrane. The other bands are unspecific binding which could be due to the many different cell types in the cerebral arteries. (B) According to Sigma, β-ACTIN was detected at 42 kDa. The membrane was stripped before incubation with β-ACTIN but there might a vague LOX1 binding left on the membrane. We used LOX1 and β-ACTIN antibodies from two different species.
(TIF)

S1 Table Information about the 169 differentially expressed genes (q = 0). (DOCX)

S2 Table Information of genes verified by qPCR. (DOCX)
