# Supplementary figures and images for: Cerebrovascular gene expression in spontaneously hypertensive rats
Source: PLoS One. 2017 Sep 7;12(9):e0184233. doi: 10.1371/journal.pone.0184233 (PMC5589213; doi:10.1371/journal.pone.0184233)

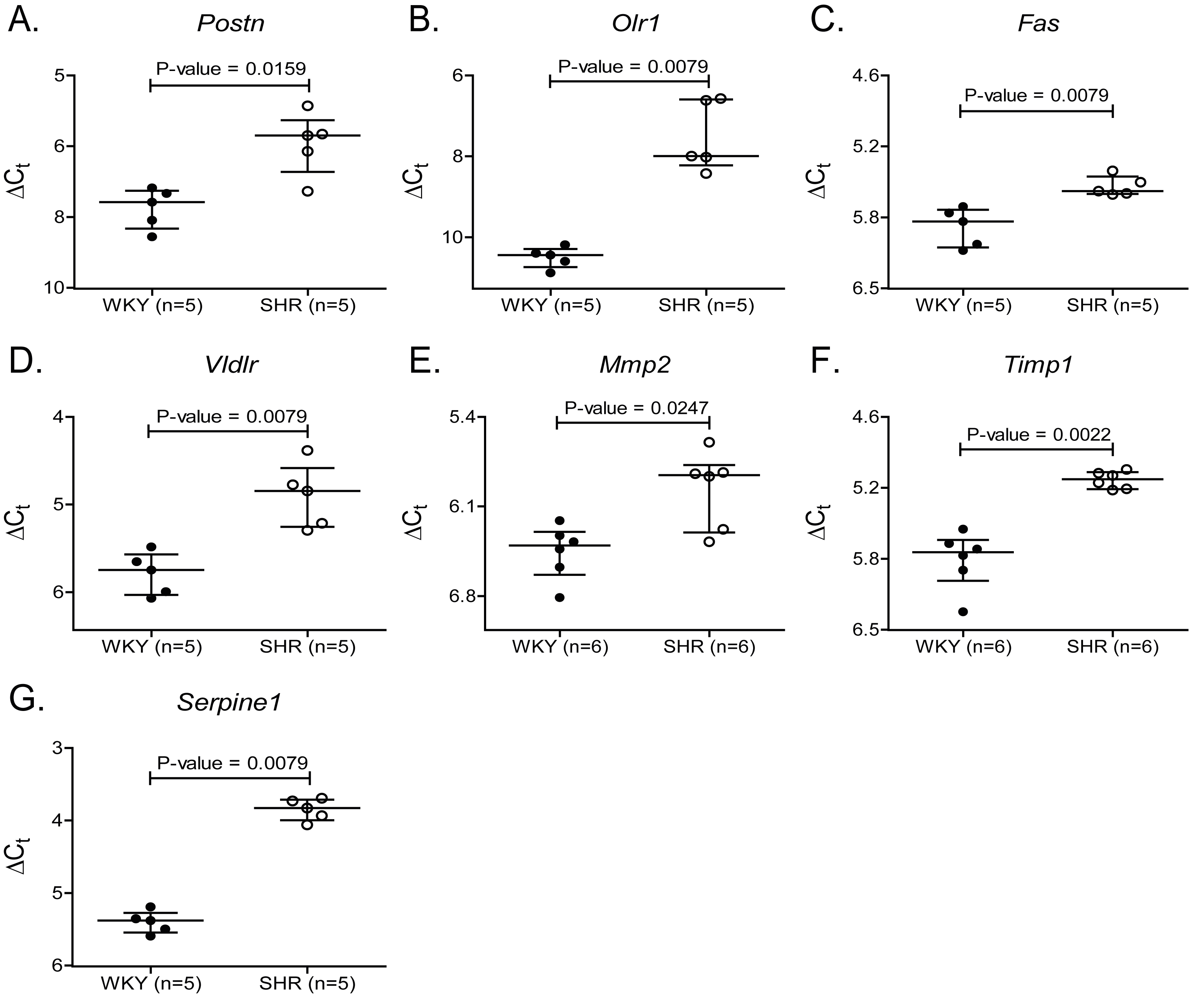

Supplement: S1 Fig — Scatter plot of (A) Postn, (B) Olr1, (C) Fas, (D) Vldlr, (E) Mmp2, (F) Timp1 and (G) Serpine 1 expression from SHRs and WKY rats. ΔCt values are plotted on the y-axe by a logarithmic scale. Data is expressed as median ± interquartile range, and n represents the number of rats. P-value <0.05 is considered statistical significant. (TIF) [file pone.0184233.s001.tif]

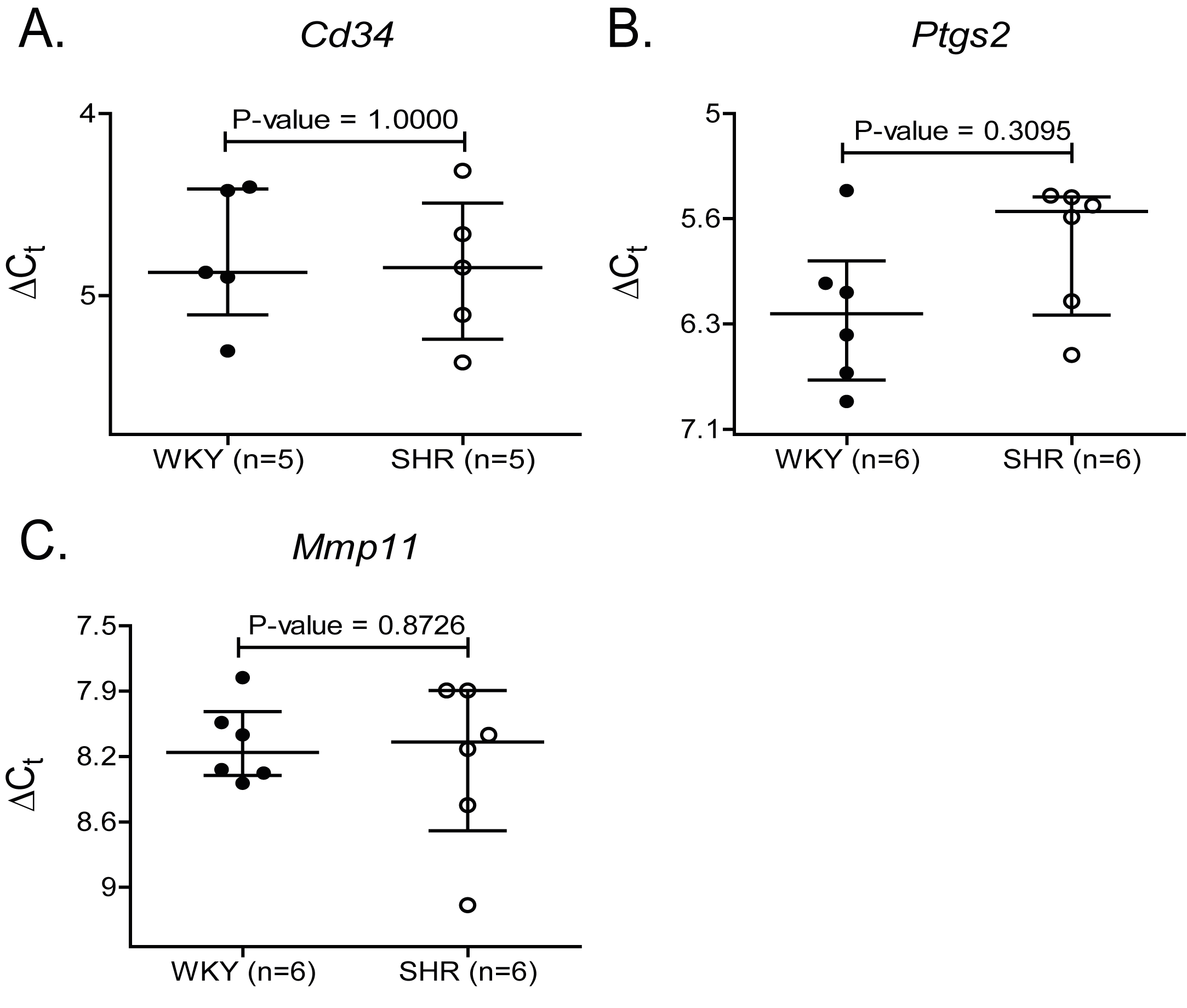

Supplement: S2 Fig — Scatter plot of (A) Cd34, (B) Ptgs2 and (C) Mmp11 expression in middle cerebral arteries from SHRs and WKY rats. ΔCt values are plotted on the y-axe by a logarithmic scale. Data is expressed as median ± interquartile range, and n represents the number of rats. P-value <0.05 is considered statistical significant. (TIF) [file pone.0184233.s002.tif]

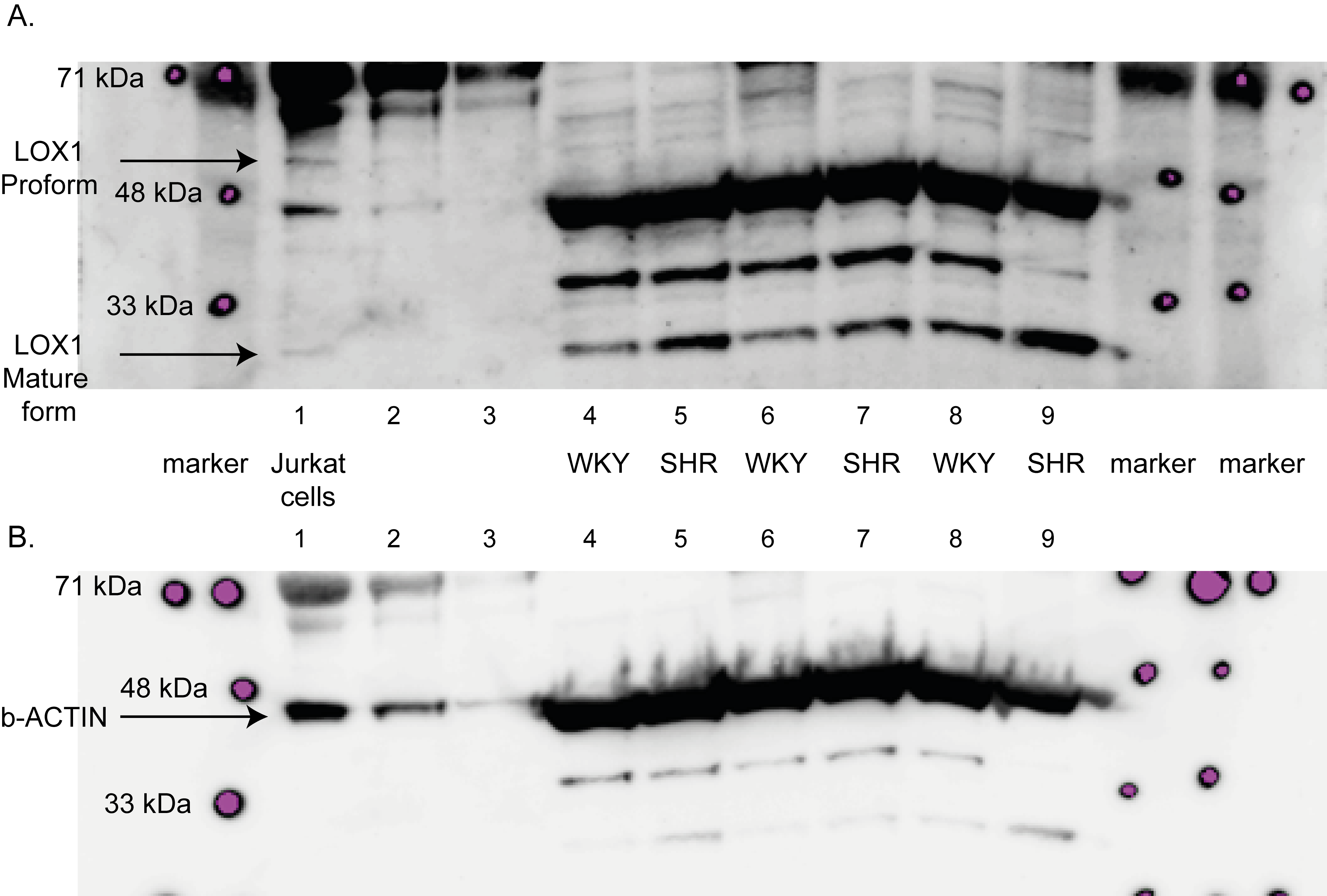

Supplement: S3 Fig — Western blot membrane with (A) LOX1 and (B) β-ACTIN. The molecular marker is marked with a black square. Before incubation with primary antibodies the membrane was cut just before 71 kDa and 28 kDa. Sample in lane 2 and 3 are samples used to test different antibodies for the other membrane pieces. (A) According to Abcam, LOX1 proform was detected at 50 kDa and LOX1 mature form was detected at 31 kDa. However, the LOX1 proform in the antibody images from Abcam homepage seems to be at 45 kDa and not 50 kDa according to the molecular marker which correlate with the band around 48 kDa on our membrane. The other bands are unspecific binding which could be due to the many different cell types in the cerebral arteries. (B) According to Sigma, β-ACTIN was detected at 42 kDa. The membrane was stripped before incubation with β-ACTIN but there might a vague LOX1 binding left on the membrane. We used LOX1 and β-ACTIN antibodies from two different species. (TIF) [file pone.0184233.s003.tif]
