# Supplementary material for: Cerebrovascular gene expression in spontaneously hypertensive rats
Source: PLoS One. 2017 Sep 7;12(9):e0184233. doi: 10.1371/journal.pone.0184233 (PMC5589213; doi:10.1371/journal.pone.0184233)
Supplement: S1 Table — (DOCX) [file pone.0184233.s004.docx]

| **Gene** | **Description** | **Gene accession no.** | **FD** |
| --- | --- | --- | --- |
| Ifit1 | interferon-induced protein with tetratricopeptide repeats 1 | NM_020096 | 11.120 |
| Chad | chondroadherin | ENSRNOT00000004435 | 4.176 |
| Fcrls | Fc receptor-like S, scavenger receptor | NM_001107702 | 3.495 |
| RT1-CE4 | RT1 class I, locus CE4 | NM_001008842 | 3.017 |
| Gad1 | glutamate decarboxylase 1 | ENSRNOT00000000008 | 2.919 |
| Postn | periostin, osteoblast specific factor | NM_001108550 | 2.635 |
| Micb | MHC class I polypeptide-related sequence B | NM_001017468 | 2.549 |
| Gpm6a | glycoprotein m6a | NM_178105 | 2.544 |
| RT1-CE12 | RT1 class I, locus CE12 | NM_001008835 | 2.532 |
| Tarsl2 | threonyl-tRNA synthetase-like 2 | NM_001014020 | 2.498 |
| Clec2l | C-type lectin domain family 2. member L | NM_001044233 | 2.495 |
| Cilp | cartilage intermediate layer protein, nucleotide pyrophosphohydrolase | ENSRNOT00000044887 | 2.216 |
| Olr1 | oxidized low density lipoprotein (lectin-like) receptor 1 | NM_133306 | 2.209 |
| Penk | proenkephalin | NM_017139 | 2.155 |
| Trpm8 | transient receptor potential cation channel, subfamily M, member 8 | NM_134371 | 2.135 |
| Slc11a1 | solute carrier family 11 (proton-coupled divalent metal ion transporters), member 1 | NM_001031658 | 2.126 |
| Kcns3 | potassium voltage-gated channel, delayed-rectifier, subfamily S, member 3 | NM_031778 | 2.116 |
| Slfn13 | schlafen family member 13 | NM_001013970 | 2.110 |
| Inmt | indolethylamine N-methyltransferase | NM_001109022 | 2.084 |
| RGD1563903 | hypothetical gene supported by X60212 | ENSRNOT00000050309 | 2.061 |
| Dfnb31 | deafness, autosomal recessive 31 | ENSRNOT00000002309 | 2.004 |
| Minos1 | mitochondrial inner membrane organizing system 1 | NM_001173556 | 1.987 |
| Scube3 | signal peptide, CUB domain, EGF-like 3 | ENSRNOT00000000594 | 1.961 |
| Fap | fibroblast activation protein, alpha | ENSRNOT00000008148 | 1.957 |
| Crispld1 | cysteine-rich secretory protein LCCL domain containing 1 | NM_001134963 | 1.909 |
| Atf3 | activating transcription factor 3 | NM_012912 | 1.901 |
| Ppapdc1a | phosphatidic acid phosphatase type 2 domain containing 1A | NM_001191631 | 1.889 |
| Clic2 | chloride intracellular channel 2 | NM_001009651 | 1.889 |
| Layn | layilin | NM_001191997 | 1.885 |
| Efcab1 | EF hand calcium binding domain 1 | NM_001106930 | 1.867 |
| Serpine1 | serpin peptidase inhibitor, clade E (nexin, plasminogen activator inhibitor type 1), member 1 | ENSRNOT00000001916 | 1.835 |
| Ryr3 | ryanodine receptor 3 | AF130881 | 1.820 |
| Fgf7 | fibroblast growth factor 7 | ENSRNOT00000012700 | 1.781 |
| Serpind1 | serpin peptidase inhibitor, clade D (heparin cofactor), member 1 | ENSRNOT00000031819 | 1.772 |
| Psmb8 | proteasome (prosome, macropain) subunit, beta type 8 (large multifunctional peptidase 7) | NM_080767 | 1.721 |
| Ptgs2 | prostaglandin-endoperoxide synthase 2 | ENSRNOT00000003567 | 1.716 |
| Dmp1 | dentin matrix acidic phosphoprotein 1 | NM_203493 | 1.700 |
| Kif15 | kinesin family member 15 | ENSRNOT00000035119 | 1.694 |
| Erbb4 | v-erb-a erythroblastic leukemia viral oncogene homolog 4 (avian) | ENSRNOT00000019283 | 1.681 |
| Clic5 | chloride intracellular channel 5 | NM_053603 | 1.670 |
| P2rx4 | purinergic receptor P2X, ligand-gated ion channel 4 | ENSRNOT00000001752 | 1.665 |
| Cd34 | CD34 molecule | NM_001107202 | 1.658 |
| Trim59 | tripartite motif-containing 59 | ENSRNOT00000013987 | 1.650 |
| Ephb1 | Eph receptor B1 | NM_001104528 | 1.631 |
| RGD1562351 | similar to chromosome 7 open reading frame 23 | BC162037 | 1.624 |
| Ifitm1 | interferon induced transmembrane protein 1 | ENSRNOT00000005645 | 1.597 |
| Cybasc3 | cytochrome b, ascorbate dependent 3 | NM_001014164 | 1.591 |
| Timp1 | TIMP metallopeptidase inhibitor 1 | NM_053819 | 1.588 |
| Abca9 | ATP-binding cassette, subfamily A (ABC1), member 9 | ENSRNOT00000005775 | 1.583 |
| Stxbp6 | syntaxin binding protein 6 (amisyn) | NM_001191872 | 1.578 |
| Ms4a11 | membrane-spanning 4-domains, subfamily A, member 11 | ENSRNOT00000033795 | 1.577 |
| LOC100366030 | rCG37858-like | NM_001177909 | 1.575 |
| Mpa2l | macrophage activation 2 like | ENSRNOT00000040985 | 1.574 |
| Shc3 | SHC (Src homology 2 domain containing) transforming protein 3 | NM_001105743 | 1.571 |
| Pank2 | pantothenate kinase 2 (Hallervorden-Spatz syndrome) | NM_001106513 | 1.570 |
| Mmp11 | matrix metallopeptidase 11 | ENSRNOT00000031400 | 1.553 |
| Ptgs1 | prostaglandin-endoperoxide synthase 1 | ENSRNOT00000010218 | 1.553 |
| Fas | Fas (TNF receptor superfamily, member 6) | ENSRNOT00000049807 | 1.550 |
| Aif1l | allograft inflammatory factor 1-like | NM_001108578 | 1.545 |
| Cygb | cytoglobin | NM_130744 | 1.541 |
| Acaa2 | acetyl-CoA acyltransferase 2 | ENSRNOT00000067018 | 1.537 |
| Mrpl18 | mitochondrial ribosomal protein L18 | NM_001106205 | 1.537 |
| RGD1561636 | similar to 60S ribosomal protein L38 | ENSRNOT00000049811 | 1.536 |
| Pcsk6 | proprotein convertase subtilisin/kexin type 6 | ENSRNOT00000015845 | 1.533 |
| Olr63 | olfactory receptor 63 | NM_001000542 | 1.498 |
| MGC105649 | hypothetical LOC302884 | NM_001008518 | 1.496 |
| Csrp2 | cysteine and glycine-rich protein 2 | NM_177425 | 1.495 |
| Kcnip2 | Kv channel-interacting protein 2 | NM_020094 | 1.490 |
| N6amt1 | N-6 adenine-specific DNA methyltransferase 1 (putative) | NM_001191592 | 1.483 |
| Serpine2 | serpin peptidase inhibitor, clade E, member 2 | NM_019197 | 1.482 |
| RGD1305464 | similar to human chromosome 15 open reading frame 39 | BC089935 | 1.477 |
| Smad9 | SMAD family member 9 | ENSRNOT00000000102 | 1.476 |
| Rpl38 | ribosomal protein L38 | NM_001077592 | 1.461 |
| Msrb2 | methionine sulfoxide reductase B2 | NM_001031660 | 1.473 |
| Sec16b | SEC16 homolog B (S. cerevisiae) | NM_053571 | 1.472 |
| Myeov2 | myeloma overexpressed 2 | NM_001109044 | 1.470 |
| Rpl38 | ribosomal protein L38 | FQ228832 | 1.469 |
| Btbd3 | BTB (POZ) domain containing 3 | NM_001107782 | 1.466 |
| Mmp2 | matrix metallopeptidase 2 | NM_031054 | 1.465 |
| Rpl38 | ribosomal protein L38 | NM_001077592 | 1.474 |
| Vldlr | very low density lipoprotein receptor | NM_013155 | 1.440 |
| Prrg4 | proline rich Gla (G-carboxyglutamic acid) 4 (transmembrane) | NM_001109203 | 1.431 |
| Leprel4 | leprecan-like 4 | NM_021581 | 1.430 |
| Tmed9 | transmembrane emp24 protein transport domain containing 9 | NM_001009703 | 1.420 |
| Art3 | ADP-ribosyltransferase 3 | NM_001012034 | 1.413 |
| Amigo2 | adhesion molecule with Ig like domain 2 | NM_182816 | 1.404 |
| Apool | apolipoprotein O-like | NM_001014105 | 1.403 |
| Gda | guanine deaminase | ENSRNOT00000024775 | 1.402 |
| LOC680353 | similar to 60S ribosomal protein L38 | ENSRNOT00000048963 | 1.402 |
| Pop5 | processing of precursor 5, ribonuclease P/MRP subunit (S. cerevisiae) | NM_001105752 | 1.399 |
| Itga1 | integrin, alpha 1 | NM_030994 | 1.396 |
| Asns | asparagine synthetase | ENSRNOT00000010079 | 1.394 |
| Fam129a | family with sequence similarity 129, member A | NM_022242 | 1.393 |
| Tspan7 | tetraspanin 7 | NM_001108815 | 1.379 |
| Slc41a2 | solute carrier family 41, member 2 | NM_001108742 | 1.369 |
| Fgd4 | FYVE, RhoGEF and PH domain containing 4 | ENSRNOT00000002491 | 1.361 |
| Acadl | acyl-CoA dehydrogenase, long chain | NM_012819 | 1.358 |
| Tpst1 | tyrosylprotein sulfotransferase 1 | NM_001011903 | 1.356 |
| Cybrd1 | cytochrome b reductase 1 | NM_001011954 | 1.343 |
| RGD1565648 | similar to Chain A, Solution Structure Of Rabbit Apo-S100a11 (19 Models) | ENSRNOT00000055510 | -17.260 |
| Slc5a5 | solute carrier family 5 (sodium iodide symporter), member 5 | NM_052983 | -6.438 |
| LOC100362296 | protein S100-A11-like | ENSRNOT00000055513 | -6.133 |
| MGC105567 | similar to cDNA sequence BC023105 | BC093614 | -4.297 |
| LOC100910367 | uncharacterized LOC100910367 | XM_003751843 | -4.170 |
| RT1-Bb | RT1 class II, locus Bb | NM_001004084 | -3.972 |
| Cxcl13 | chemokine (C-X-C motif) ligand 13 | NM_001017496 | -3.433 |
| Il33 | interleukin 33 | NM_001014166 | -3.042 |
| Hba-a1 | globin, alpha | ENSRNOT00000052292 | -2.613 |
| Cidea | cell death-inducing DFFA-like effector a | NM_001170467 | -2.607 |
| Sh2d3c | SH2 domain containing 3C | NM_001108579 | -2.579 |
| Chrdl1 | kohjirin | NM_199502 | -2.552 |
| Hbb-b1 | hemoglobin, beta adult major chain | ENSRNOT00000019913 | -2.517 |
| A2ld1 | AIG2-like domain 1 | NM_001025634 | -2.444 |
| LOC302022 | similar to nidogen 2 protein | BC162000 | -2.360 |
| Ckm | creatine kinase, muscle | ENSRNOT00000022895 | -2.345 |
| Wdr46 | WD repeat domain 46 | NM_212491 | -2.221 |
| Insrr | insulin receptor-related receptor | NM_022212 | -2.191 |
| Acss1 | acyl-CoA synthetase short-chain family member 1 | NM_001106524 | -2.171 |
| MGC108823 | similar to interferon-inducible GTPase | NM_001012353 | -2.073 |
| Mrps10 | mitochondrial ribosomal protein S10 | NM_001008859 | -2.062 |
| Myoz2 | myozenin 2 | NM_001106469 | -2.018 |
| Rpl30 | ribosomal protein L30 | K02932 | -1.988 |
| St6galnac2 | ST6 (alpha-N-acetyl-neuraminyl-2,3-beta-galactosyl-1,3)-N-acetylgalactosaminide alpha-2,6-sialyltransferase 2 | ENSRNOT00000016622 | -1.966 |
| Ifi44l | interferon-induced protein 44-like | XM_227820 | -1.874 |
| Acadsb | acyl-CoA dehydrogenase, short/branched chain | NM_013084 | -1.821 |
| Hpgds | hematopoietic prostaglandin D synthase | NM_031644 | -1.816 |
| Cyp26b1 | cytochrome P450, family 26, subfamily b, polypeptide 1 | NM_181087 | -1.811 |
| Retsat | retinol saturase (all trans retinol 13,14 reductase) | ENSRNOT00000019571 | -1.794 |
| Bdh1 | 3-hydroxybutyrate dehydrogenase, type 1 | NM_053995 | -1.778 |
| Hist1h2bc | histone cluster 1, H2bc | NM_001109400 | -1.764 |
| C8g | complement component 8, gamma polypeptide | NM_001106555 | -1.746 |
| Bmp5 | bone morphogenetic protein 5 | NM_001108168 | -1.739 |
| Tmem18 | transmembrane protein 18 | NM_001007748 | -1.729 |
| Sema3c | sema domain, immunoglobulin domain (Ig), short basic domain, secreted, (semaphorin) 3C | NM_001106578 | -1.723 |
| Pdxk | pyridoxal (pyridoxine, vitamin B6) kinase | ENSRNOT00000001589 | -1.691 |
| Adgrd1 | similar to G protein-coupled receptor 133 | ENSRNOT00000058586 | -1.678 |
| Faim3 | Fas apoptotic inhibitory molecule 3 | NM_001014843 | -1.674 |
| Isyna1 | inositol-3-phosphate synthase 1 | NM_001013880 | -1.670 |
| Dctd | dCMP deaminase | NM_001161512 | -1.663 |
| Mrpl4 | mitochondrial ribosomal protein L4 | NM_001108754 | -1.625 |
| Tti1 | TELO2 interacting protein 1 | NM_001134619 | -1.620 |
| Crot | carnitine O-octanoyltransferase | ENSRNOT00000067462 | -1.608 |
| Sema5b | sema domain, seven thrombospondin repeats (type 1 and type 1-like), transmembrane domain (TM) and short cytoplasmic domain, (semaphorin) 5B | NM_001107091 | -1.607 |
| Pkp2 | plakophilin 2 | ENSRNOT00000002498 | -1.606 |
| Sulf1 | sulfatase 1 | ENSRNOT00000012610 | -1.598 |
| Itga11 | integrin, alpha 11 | NM_001108156 | -1.598 |
| Pnoc | prepronociceptin | NM_013007 | -1.595 |
| LOC679748 | similar to Macrophage migration inhibitory factor (MIF) (Phenylpyruvate tautomerase) (Glycosylation-inhibiting factor) (GIF) (Delayed early response protein 6) (DER6) | XM_001054317 | -1.571 |
| Syndig1 | synapse differentiation inducing 1 | NM_001025020 | -1.567 |
| Gper | G protein-coupled estrogen receptor 1 | NM_133573 | -1.566 |
| Ppp1r16b | protein phosphatase 1, regulatory subunit 16B | NM_001191072 | -1.553 |
| Olr1448 | olfactory receptor 1448 | NM_001000019 | -1.548 |
| Mettl24 | hypothetical protein LOC499465 | NM_001025038 | -1.544 |
| Ncln | nicalin | NM_001014082 | -1.544 |
| Fmod | fibromodulin | NM_080698 | -1.544 |
| Aplf | aprataxin and PNKP like factor | NM_001173382 | -1.517 |
| H2afx | H2A histone family, member X | NM_001109291 | -1.514 |
| Bcl6b | B-cell CLL/lymphoma 6, member B | NM_001108279 | -1.513 |
| Stard6 | StAR-related lipid transfer (START) domain containing 6 | NM_001007627 | -1.512 |
| F2rl1 | coagulation factor II (thrombin) receptor-like 1 | NM_053897 | -1.503 |
| Maf | v-maf musculoaponeurotic fibrosarcoma oncogene homolog (avian) | NM_019318 | -1.502 |
| Fam115c | family with sequence similarity 115, member C | ENSRNOT00000050392 | -1.496 |
| Tspan18 | tetraspanin 18 | NM_001107750 | -1.485 |
| Isca1 | iron-sulfur cluster assembly 1 homolog (S. cerevisiae) | NM_181626 | -1.477 |
| C1qtnf3 | C1q and tumor necrosis factor related protein 3 | NM_001134436 | -1.472 |
| Chn2 | chimerin (chimaerin) 2 | ENSRNOT00000012655 | -1.463 |
| Susd4 | sushi domain containing 4 | NM_001105982 | -1.424 |
| Elovl7 | ELOVL fatty acid elongase 7 | ENSRNOT00000014074 | -1.421 |
| Lrsam1 | leucine rich repeat and sterile alpha motif containing 1 | NM_001107833 | -1.400 |
| Clmn | calmin | NM_001106755 | -1.396 |
| Lss | lanosterol synthase (2,3-oxidosqualene-lanosterol cyclase) | ENSRNOT00000042499 | -1.395 |

FD, fold difference.
