# Supplementary material for: Cerebrovascular gene expression in spontaneously hypertensive rats
Source: PLoS One. 2017 Sep 7;12(9):e0184233. doi: 10.1371/journal.pone.0184233 (PMC5589213; doi:10.1371/journal.pone.0184233)
Supplement: S2 Table — (DOCX) [file pone.0184233.s005.docx]

| **Gene** | **Description** | **Gene accession no.** | **FD** |
| --- | --- | --- | --- |
| *Postn* | Periostin | NM_001108550 | 2.634 |
| *Olr1* | Oxidized low density lipoprotein (lectin-like) receptor 1 | NM_133306 | 2.208 |
| *Serpine1* | Serpin peptidase inhibitor, clade E (nexin, plasminogen activator inhibitor type 1), member 1 | ENSRNOT00000001916 | 1.835 |
| *Cd34* | Cd34 molecule | NM_001107202 | 1.658 |
| *Timp1* | Tissue inhibitor metallopeptidase 1 | NM_053819 | 1.588 |
| *Fas* | Fas (TNF receptor superfamily, member 6) | ENSRNOT00000049807 | 1.550 |
| *Mmp2* | Matrix metallopeptidase 2 | NM_031054 | 1.456 |
| *Vldlr* | Very low density lipoprotein receptor | NM_013155 | 1.440 |
| *Ptgs1* | Prostaglandin-endoperoxide synthase 1 | ENSRNOT00000010218 | 1.552 |
| *Ptgs2* | Prostaglandin-endoperoxide synthase 2 | ENSRNOT00000003567 | 1.716 |
| *Mmp11* | Matrix metallopeptidase 11 | ENSRNOT00000031400 | 1.553 |

FD, fold difference.
